# Development and Validation of Response Time Inconsistency in the Midlife in the United States (MIDUS) Study

**DOI:** 10.1093/geroni/igaf122.2407

**Published:** 2025-12-31

**Authors:** Robert Stawski, Eric Cerino, Dakota Witzel, Margie Lachman, Stuart MacDonald

**Affiliations:** Utah State University, Logan, Utah, United States; Northern Arizona University, Flagstaff, Arizona, United States; South Dakota State University, Brookings, South Dakota, United States; Brandeis University, Waltham, Massachusetts, United States; University of Victoria, Victoria, British Columbia, Canada

## Abstract

Response time inconsistency (RTI), the trial-to-trial variability on a RT-based cognitive task, is an important behavioral indicator of cognitive health, cognitive aging, and an early indicator of cognitive pathology, and central nervous system (CNS) dysfunction. Although RTI is well-studied through computer-based speeded tasks, little research has examined the validity of RTI obtained from: (1) telephone-based cognitive assessments employing voice-triggered response time protocols; or (2) midlife cohorts who are at risk for dementia. Using data from the second wave of the MIDUS study (N = 4,285; Mage=55.6, SD = 12.2, Range=28-84; 55%=women; 75% having at least some college education), participants completed the Brief Test of Adult Cognition via Telephone (BTACT), which includes tests of executive function and episodic memory abilities, as well as a RT-based stop-go switching (SGS) task. RTI was derived from 20 trials of the SGS non-switch condition and quantified as an intraindividual standard deviation (ISD) residualized for trial number. RTI was greater with age (r=.26, p<.0001), being less educated (r=-.16, p<.0001), and among women (Cohen’s d=.15, p<.0001). Importantly, RTI was associated with slower task switching performance, lower scores on the BTACT, episodic memory, and executive function cognitive composites (rs=-.19 to -.41, ps<.0001), as well as poorer self-rated mental (r=-.13) and physical (-.18, ps<.0001) health. RTI was not significantly related to self-rated positive affect (r=-.02, p=.24) or negative affect (r=.02, p=.17). Discussion will focus on the validity and promise of RTI obtained from telephone-based cognitive assessments for characterizing CNS integrity and dementia risk in large-scale surveys.

